# Prognostic Roles of Central Carbon Metabolism–Associated Genes in Patients With Low-Grade Glioma

**DOI:** 10.3389/fgene.2019.00831

**Published:** 2019-09-18

**Authors:** Li Wang, Meng Guo, Kai Wang, Lei Zhang

**Affiliations:** ^1^Department of Neurosurgery, Xijing Hospital, Fourth Military Medical University, Xi’an, China; ^2^Xijing Hospital of Digestive Diseases, Fourth Military Medical University, Xi’an, China

**Keywords:** low-grade glioma, astrocytoma, prognosis, metabolism, gene expression

## Abstract

**Purpose:** Metabolic alterations are crucial for tumor progression and response to therapy. The comprehensive model of combined central carbon metabolism–associated genes that contribute to the outcomes of glioma and astrocytoma is not well understood.

**Method:** We studied the profiles of 63 genes involved in central carbon metabolism in 514 relatively low-grade glioma patients. The different distributions of gene expression in gliomas and astrocytoma were identified. The differential gene expression between each cohort and the correlations with prognosis were detected. Finally, we built a tentative model to detect the prognostic roles of carbon metabolism–associated genes in astrocytoma.

**Result:** Two primary clusters and four subclusters with significantly different overall survival were identified in low-grade glioma. The differences of histological diagnoses, grade, tumor site, and age were detected between each cluster. Comparing with other histological types, patients with astrocytoma exhibited the worst prognosis. Between astrocytoma patients with poor and favorable prognoses, expression profiles of 11 genes were significantly discrepant. We detected that 18 genes were respectively correlated with overall survival in astrocytoma; moreover, four genes (*RAF1*, *AKT3*, *IDH1*, and *FGFR1*) were detected as dependent variables for the prediction of the survival status of astrocytoma patients and were capable to predict the survival.

**Conclusion:** Central carbon metabolism–associated genes are differentially expressed in all patients with glioma and histological subtype astrocytoma. The gene expression profile is significantly associated with clinical manifestations. These results suggested that both the multigene expression patterns and individual central carbon metabolism–associated genes were potentially capable to predict the prognosis of patients with low-grade glioma.

## Introduction

Diffuse low-grade gliomas are the most common primary malignancies in adults and include astrocytomas, oligodendrogliomas, and oligoastrocytomas (Brat et al., 2015). Different histological subtypes of glioma were undistinguishable; however, large differences in clinical behavior and response to therapy suggest that difference among the histological types is crucial (Smith et al., 2000). Even within each subtype, there are large differences in clinical performance among individual patients. Surgery resection is the primary therapeutic method for low-grade gliomas, but the outcomes are less than satisfactory because of the highly infiltrative nature of glioma, and the presence of residual tumor tissue results in recurrence and malignant progression (Dixit and Raizer, 2017). The prognosis of patients with relatively low-grade glioma varies widely, with some patients living for more than 5 years, while others survive less than 1 year (Bush and Chang, 2016). A more precise method of predicting the outcomes of relatively low-grade glioma is urgently needed to be developed.

Metabolic reprogramming is a central hallmark of cancer. Dysregulation of metabolism-related genes leads to cellular transformation and tumor progression. Warburg (1956) revealed differences in the central metabolic pathways in solid tumors and noted that cancer cells require a large amount of glucose to maintain a high rate of glycolysis even in the presence of adequate oxygen and that they convert a majority of that glucose into lactic acid (the Warburg effect). More recently, it has been recognized that the “Warburg effect” contains a similarly increased utilization of glutamine (Reitzer et al., 1979). Previous studies have detected some variations in the genes, such as *IDH1/2*, *GLUT1*, and *GLUT3*, involved in tumor metabolism in gliomas (Yan et al., 2009; Verhaak et al., 2010; Labak et al., 2016). High-throughput sequencing has substantially advanced the understanding of the metabolic changes in low-grade gliomas by detecting changes in metabolism-associated genes (Brennan et al., 2013). Profiling holistic gene expression not only facilitates the investigation of subgroups with low-grade glioma but also enables the identification of the predictors of overall survival (OS) (Chen et al., 2016). Which pattern of expression of metabolism-associated genes in tumor tissue contributes to glioma is not well understood. The Cancer Genome Atlas (TCGA) provided a standardized gene expression dataset for the study of the expression pattern of metabolism-related genes, which enables the investigation of correlations between clinical manifestations and carbon metabolism–associated genes in glioma (The Cancer Genome Atlas Research Network et al., 2008; Sanborn et al., 2013).

In this study, we investigated the expression patterns of central carbon metabolism–associated genes in adult patients with diffuse low-grade glioma, including astrocytoma, oligodendroglioma, and oligoastrocytoma. Moreover, we respectively detected the prognostic roles of individual gene and the multiple-gene combination. These results will facilitate an integral understanding of the metabolic alterations in glioma and provide a novel perspective to manage and treat this lethal cancer.

## Methods

### Samples and Database

We obtained transcriptome data and the corresponding clinical data of 514 relatively low-grade glioma patients from TCGA from the cBioPortal for Cancer Genomics (http://cbioportal.org) (Gao et al., 2013). We filtered the data based on whether the mRNA *z*-score data, histological diagnosis, and OS data were comprehensive. Collectively, the studied dataset included 194 astrocytoma samples, 130 oligoastrocytoma samples, and 190 oligodendroglioma samples.

Central carbon metabolism–related genes in the cancer-associated gene panel (hsa05230) were derived from the KEGG pathway database (http://www.kegg.jp/kegg/), as previously described (Kanehisa et al., 2017). In total, 65 central carbon metabolism–associated genes were listed; however, transcriptome information was missing for *MYC* and *HKDC1*, and the remaining 63 candidate genes were included after filtration. The gene expression levels were calculated from the mRNA *z* scores and compared to the expression distribution of each gene from tumors that were diploid for the genes in 514 patients with glioma (RNA-Seq V2 RSEM), based on TCGA data.

### Bioinformatics

A cluster analysis of the 63 genes expressed in each histological type was used to distinguish samples based on gene expression patterns. Samples with different gene expression patterns were identified from the whole dataset. The transcriptional levels were shown as mRNA *z* scores and clustered using the hierarchical clustering algorithm in the Gene Cluster 3.0 program (De Hoon et al., 2004). The cluster heat map and pattern according to tumor stage were generated with the Java Treeview program (Saldanha, 2004).

### Prognostic Implication Analyses

To investigate the prognostic role of the cancer metabolism–associated genes, we used GraphPad Prism 6 for Windows (GraphPad Software, Inc., CA, US; version 6.01, 2012) to perform comparisons of the overall survivals in different clusters. Additionally, an analysis of the difference in OS between the cohorts with low and high expression levels of differentially expressed genes was conducted with GraphPad Prism 6.

### Statistical Analysis

Survival curves were plotted according to the Kaplan–Meier method and compared using the log-rank test in GraphPad Prism 6. Associations between clinical characteristics and the variables used to determine the clusters of patients were analyzed by Fisher exact test and the Pearson/Spearman correlation. Differences in gene expression levels between clusters were analyzed by analysis of variance. Correlations between variable were determined by regression analyses. All tests were performed with SPSS 19.0 (IBM, Inc., NY, US). *P* < 0.05 was considered statistically significant.

## Results

### Expression Profile of Central Carbon Metabolism–Associated Genes in Diffuse Gliomas

**Figure 1:**
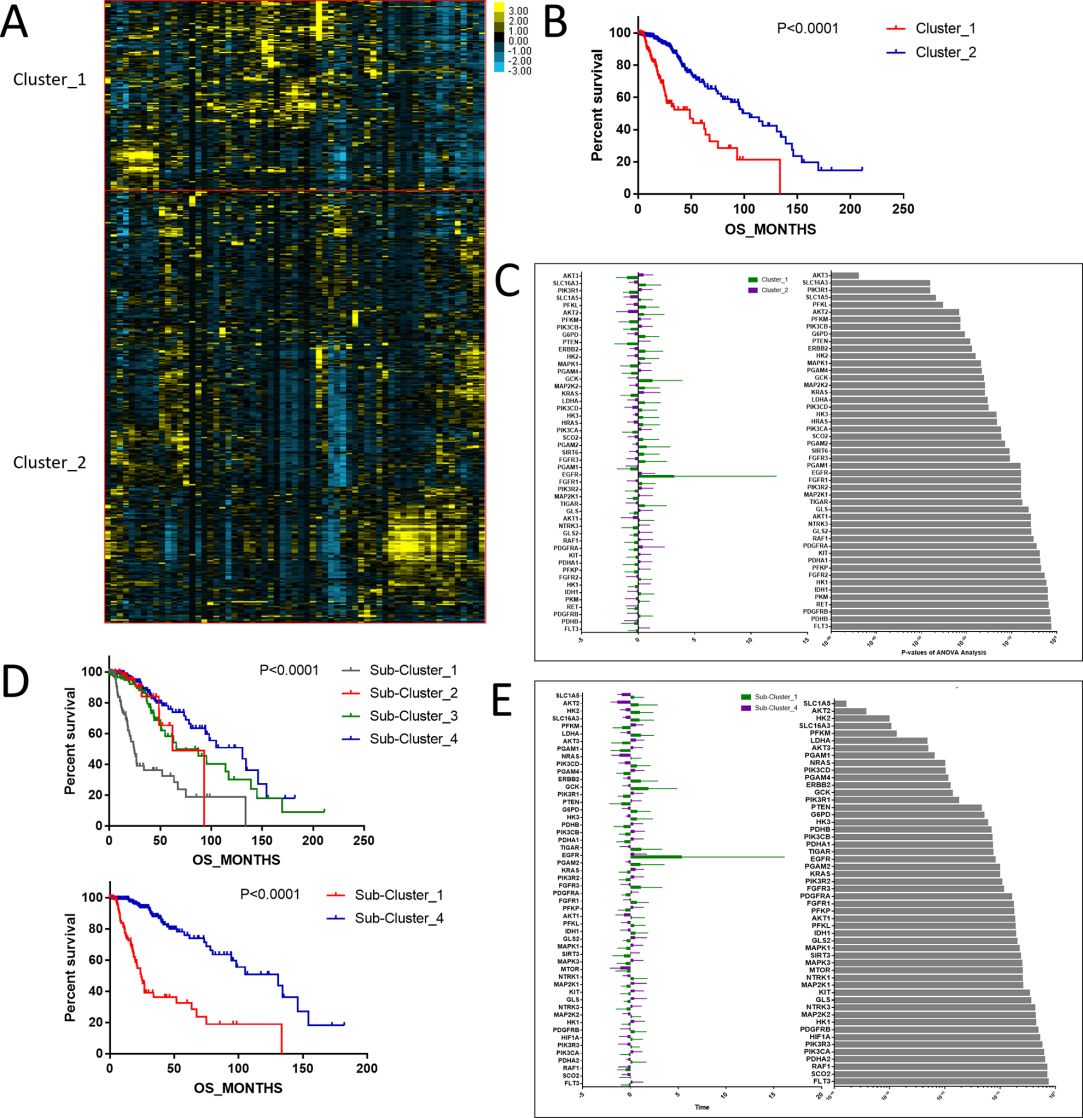
The expression profile of central carbon metabolism–associated genes in glioma patients. **(A)** In total, 514 patients were primarily divided into two clusters. The expression values of 63 genes corresponding to the individual patient were arrayed in the columns according to the expression affinity. Patients with similar gene expression patterns were clustered and grouped using the hierarchical clustering algorithm and arrayed in the rows. **(B)** The patients in cluster 1 had much worse prognoses than the patients in cluster 2, of which overall survival (OS) was 48.65 months compared to 105.12 months. **(C)** There were 49 genes that showed a significant difference in the expression levels between the two clusters *P* < 0.05. **(D)** The studied cohort was further subdivided into four subclusters, among which the subcluster 1 was with the worst OS and subcluster 4 showed the most favorable outcome. **(E)** The differential expression analysis revealed that 52 metabolism-associated genes were significantly different between subcluster 1 and subcluster 4.

To investigate central carbon metabolism programming in diffuse gliomas, we first examined the transcriptional distributions of carbon metabolism–associated genes. In total, 63 genes that have been widely reported to be key players in metabolic reprogramming were included (Soga, 2013). The patients with diffuse glioma were sorted by differences in the gene expression according to the RNA-Seq data. Following filtration, 514 patients with survival data were included in the cluster analysis (**Figure 1A**). The preliminary analysis showed that there were two clusters, and strikingly, the 101 patients in cluster 1 had much worse prognoses than the patients in cluster 2 (OS of 48.65 vs 105.12 months, *P* < 0.0001) (**Figure 1B**, **Table 1**). Between the two clusters, there was a significant difference in the expression levels (*P* < 0.05) of 49 genes (**Figure 1C**). A comparison of the clinical characteristics of clusters 1 and 2 showed that the parameters of histological diagnoses, tumor grade, tumor site, and age were vastly different between the two clusters (*P* < 0.001), as shown in **Table 2**.

**Table 1:** Overall survival differences of each cluster.

	Cluster		Significance *(P)*
	1	2	
Number	158	356	<0.0001
Median survival	48.65	105.12	

**Table 2:** Characteristics of glioma patients in clustered groups 1 and 2.

Clinical features		Cluster		Total	*P*
		1	2		
Histological diagnosis	Astrocytoma	92	102	194	2.96E−10***
	Oligoastrocytoma	34	96	130	
	Oligodendroglioma	32	158	190	
Grade	Unknown	0	1	1	<0.001***
	G2	52	196	248	
	G3	106	159	265	
Age	≤41	53	215	268	1.37E−8***
	>41	105	141	246	
Tumor site	Unknown	0	1	1	<0.001***
	Posterior fossa, brain stem	1	0	1	
	Posterior fossa, cerebellum	2	0	2	
	Supratentorial, frontal lobe	73	229	302	
	Supratentorial, not otherwise specified	7	1	8	
	Supratentorial, occipital lobe	0	8	8	
	Supratentorial, parietal lobe	18	28	46	
	Supratentorial, temporal lobe	57	89	146	
Laterality	Unknown	1	4	5	0.412
	Left	79	171	250	
	Midline	4	3	7	
	Right	74	178	252	
Supratentorial localization	Unknown	7	22	29	0.853
	Cerebral cortex	43	98	141	
	Deep gray	1	2	3	
	Not listed in medical Record	73	149	222	
	White matter	34	85	119	
Sex	Female	74	155	229	0.275
	Male	84	201	285	
History neoadjuvant	No	158	353	511	0.719
	Yes	0	3	3	

***P < 0.001.

To detect the subtler differences among further stratified cohorts, we subdivided the 514 patients into four subclusters based on the expression of the 63 genes. We detected that extreme differences in prognosis were shown among those subclusters (*P* < 0.0001) (**Figure 1D**, **Table 3**). Subcluster 1 was associated with the worst OS, while subcluster 4 showed a much favorable outcome than other subclusters (**Figure 1D**). Comparison of the gene expression variations revealed that the expression levels of 52 metabolism-associated genes were significantly different between the two prognostic-discrepant cohorts (**Figure 1E**). Additionally, we compared the clinical characteristics of the subclusters. Similar with the previous result, clear significant differences were found with regard to the parameters of histological diagnoses, tumor grade, tumor site, and age (*P* < 0.001) (**Table 4**).

**Table 3:** Overall survival differences of each subcluster

	Subcluster				Significance *(P)*
	1	2	3	4	
Number	101	57	123	233	<0.0001
Median survival	24.38	62.12	87.39	130.68	

**Table 4:** Characteristics of glioma patients in subdivided clusters.

Clinical features		Subcluster				Total	*P*
		1	2	3	4		
Histological diagnosis	Astrocytoma	67	25	60	42	194	5.94E−21***
	Oligoastrocytoma	19	15	40	56	130	
	Oligodendroglioma	15	17	23	135	190	
Grade	Unknown	0	0	0	1	1	3.88E−08***
	G2	20	32	60	136	248	
	G3	81	25	63	96	265	
Age	≤41	32	21	84	131	268	5.32E−8***
	>41	69	36	39	102	246	
Tumor site	Unknown	0	0	0	1	1	<0.001***
	Posterior fossa, brain stem	1	0	0	0	1	
	Posterior fossa, cerebellum	2	0	0	0	2	
	Supratentorial, frontal lobe	40	33	71	158	302	
	Supratentorial, not otherwise specified	4	3	0	1	8	
	Supratentorial, occipital lobe	0	0	3	5	8	
	Supratentorial, parietal lobe	11	7	15	13	46	
	Supratentorial, temporal lobe	43	14	34	55	146	
Laterality	Unknown	1	0	2	2	5	0.235
	Left	44	35	60	111	250	
	Midline	4	0	1	2	7	
	Right	52	22	60	118	252	
Supratentorial localization	Unknown	4	3	6	16	29	0.910
	Cerebral cortex	24	19	32	66	141	
	Deep gray	1	0	0	2	3	
	Not listed in medical record	50	23	55	94	222	
	White matter	22	12	30	55	119	
Sex*	Female	45	29	47	108	229	0.357
	Male	56	28	76	125	285	
History neoadjuvant	No	100	55	119	225	499	0.453
	Yes	1	2	4	8	15	

***represent P < 0.001.

### Variations in Metabolism-Associated Gene Expression Levels in Different Histological Types

**Figure 2:**
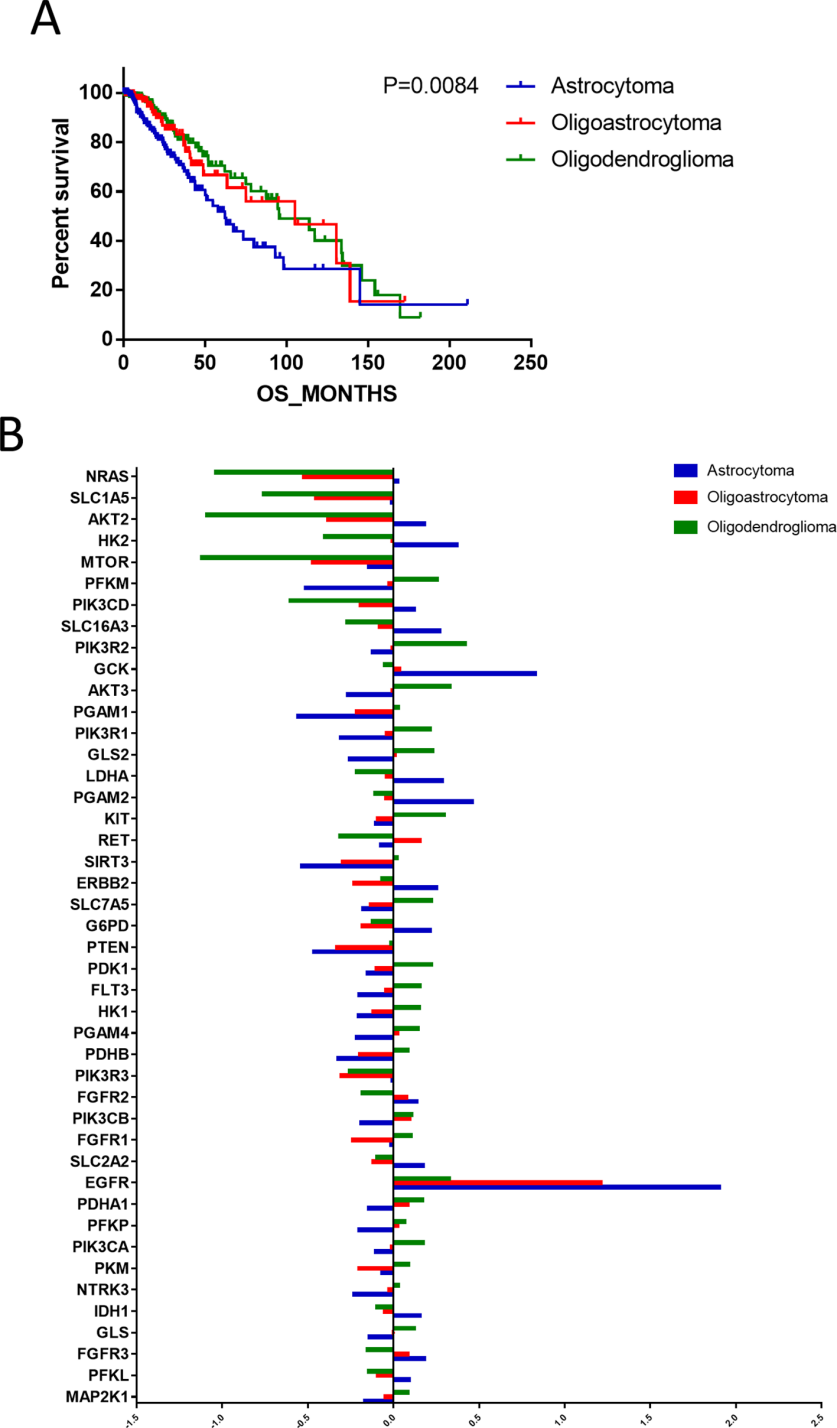
The differences in prognoses of patients with astrocytoma, oligoastrocytoma, and oligodendroglioma were significant. **(A)** The prognoses of patients with astrocytoma, oligoastrocytoma, and oligodendroglioma were significant. **(B)** Differential expression analysis demonstrated that 45 metabolism-associated genes were discrepantly expressed among the three histological types.

According to the binary comparisons among different gene expression cohorts, histological type was revealed as the variable associated with the largest differences in gene expression. We compared the OS of patients with astrocytoma, oligoastrocytoma, and oligodendroglioma, and significant differences were detected (**Figure 2A**). The median survival times of patients with astrocytoma (66.12 months), oligoastrocytoma 5.12 months), and oligodendroglioma (95.5 months) were markedly distinguishing (*P* = 0.0084). Further analysis of differences in gene expression demonstrated that 45 metabolism-associated genes were differentially expressed among the histological types (**Figure 2B**, **Supplementary Table 1**).

### Differences in the Expression Levels of Metabolism-Associated Genes in Astrocytoma

**Figure 3:**
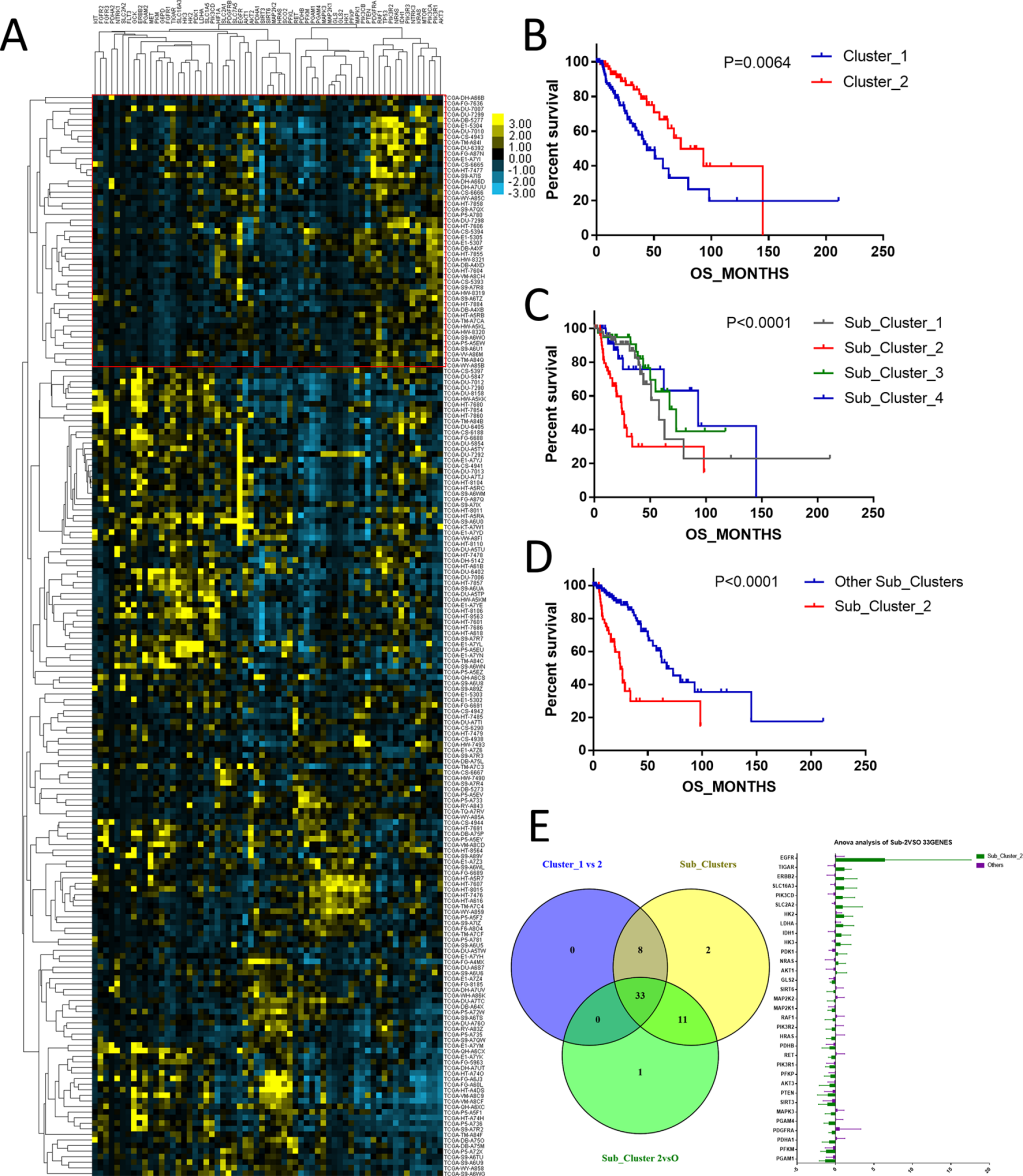
The expression profile of central carbon metabolism–associated genes in patients with astrocytoma. **(A)** According to expression profiling, two primary clusters and additionally four subdivided clusters were identified. The comparison of median survival between two primary clusters **(B)** and five subdivided clusters **(C)** showed significant difference. In addition, patients in subcluster 2 showed the worst prognosis comparing to other patients, *P* < 0.0001 **(D)**. **(E)** Differential expression analysis demonstrated that 33 metabolism-associated genes were significantly variated in all contrast of clusters 1 and 2, subcluster 2 and the other subclusters, and five subclusters.

The above results showed that among the histological types of glioma, astrocytoma showed the worst prognosis. To study the expression profiles of metabolism-associated genes in the poor-prognosis histological types, we grouped the patients with astrocytoma according to the metabolism-associated genes transcriptional data. Among the patients, two primary clusters that showed distinguishing median survival times of 43.99 and 73.42 months were identified (*P* = 0.0064) (**Figures 3A, B**). Further, four subclusters were divided according to fine grouping. The comparison of the OS showed that the difference in prognosis was even more marked (*P* < 0.0001) (**Figure 3C**). Subcluster 2 had the worse prognosis (median of 24.9 months) than other subclusters (median of 67.41 months), *P* < 0.0001 (**Figure 3D**). We respectively detected the gene expression differences between cluster 1 versus cluster 2; among different subclusters and subcluster 2 versus the other subclusters, the results revealed that the expressions of 33 metabolism-associated genes were significantly varied (**Figure 3E**).

### The Prognostic Role of Metabolism-Associated Genes in Astrocytoma

**Figure 4:**
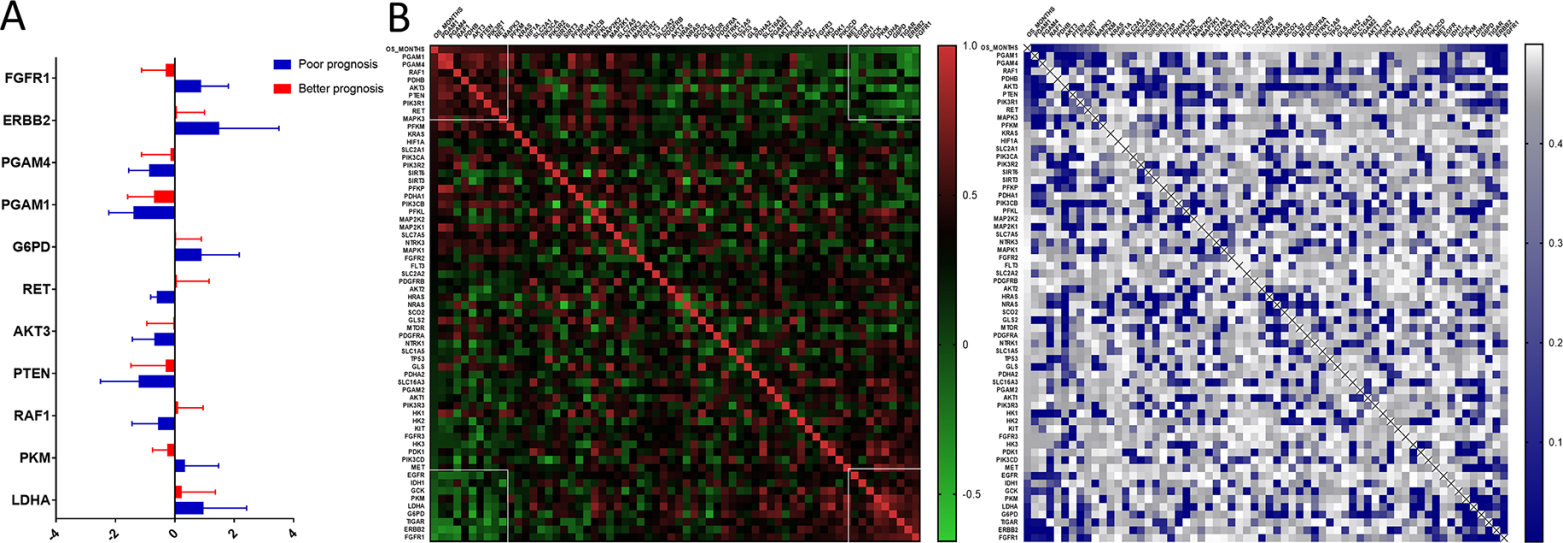
The prognostic role of metabolism-associated genes in astrocytoma. **(A)** There are 11 differentially expressed genes, containing *FGFR1*, *ERBB2*, *PGAM4*, *PGAM1*, *G6PD*, *RET*, *AKT3*, *PTEN*, *RAF1*, *PKM*, and *LDHA*, which were detected to be significantly discrepant between patients with poor and favorable overall survival (OS) times. **(B)** According to study correlation of individual gene expression and survival, the expression levels of nine genes positively correlated with OS (*r* > .2, *P* < 0.05), and the expression levels of the other nine genes were negatively correlated with OS (*r* < –0.2, *P* < 0.05).

We uncovered that the expression pattern of metabolism-associated genes was closely related to the prognosis of patients with astrocytoma. To investigate the effect of individual metabolism-associated gene on the prognosis of astrocytoma patients, we divided the subjects into two cohorts according to the OS: poor prognosis group and good prognosis group. We further investigated the differences in the expression levels of the metabolism-associated genes. It was detected that 11 genes, namely, *FGFR1*, *ERBB2*, *PGAM4*, *PGAM1*, *G6PD*, *RET*, *AKT3*, *PTEN*, *RAF1*, *PKM*, and *LDHA*, had significantly different expression levels between patients with poor and favorable OS times (**Figure 4A**, **Supplementary Table 2**).

Additionally, we detected the pertinences between the trend of metabolism-associated gene expression differential and survival variation. According to study correlation of individual gene expression and survival, positive correlations were detected between the respective expression levels of nine genes containing *PGAM1*, *PGAM4*, *RAF1*, *PDHB*, *AKT3*, *PTEN*, *PIK3R1*, *RET*, and *MAPK3* with OS (*r* > 0.2, *P* < 0.05); on the other hand, the expression levels of nine genes containing *EGFR*, *IDH1*, *GCK*, *PKM*, *LDHA*, *G6PD*, *TIGAR*, *ERBB2*, and *FGFR1* were detected negatively correlated to survival (*r* < −0.2, *P* < 0.05) (**Table 5**). In addition to their associations with survival, the expression levels of the genes are closely correlated between the two sets (**Figure 4B**).

**Table 5:** The correlation of overall survival (OS) of astrocytoma and expressing variation of individual gene.

OS positively correlated genes			OS negatively correlated genes		
Pearson correlation coefficient *(r)*		Significant*(P)*	Pearson correlation coefficient *(r)*		Significant*(P)*
PGAM1	0.41	< 0.001***	FGFR1	−0.45	<0.001***
PGAM4	0.40	0.001**	ERBB2	−0.39	0.001**
RAF1	0.36	0.003**	TIGAR	−0.37	0.002**
PDHB	0.29	0.012*	G6PD	−0.33	0.006**
AKT3	0.28	0.016*	LDHA	−0.32	0.007**
PTEN	0.27	0.020*	PKM	−0.28	0.017*
PIK3R1	0.26	0.025*	IDH1	−0.27	0.021*
RET	0.25	0.029*	GCK	−0.27	0.018*
MAPK3	0.25	0.031*	EGFR	−0.23	0.039*

*P < 0.05; **P < 0.01; ***P < 0.001.

**Figure 5:**
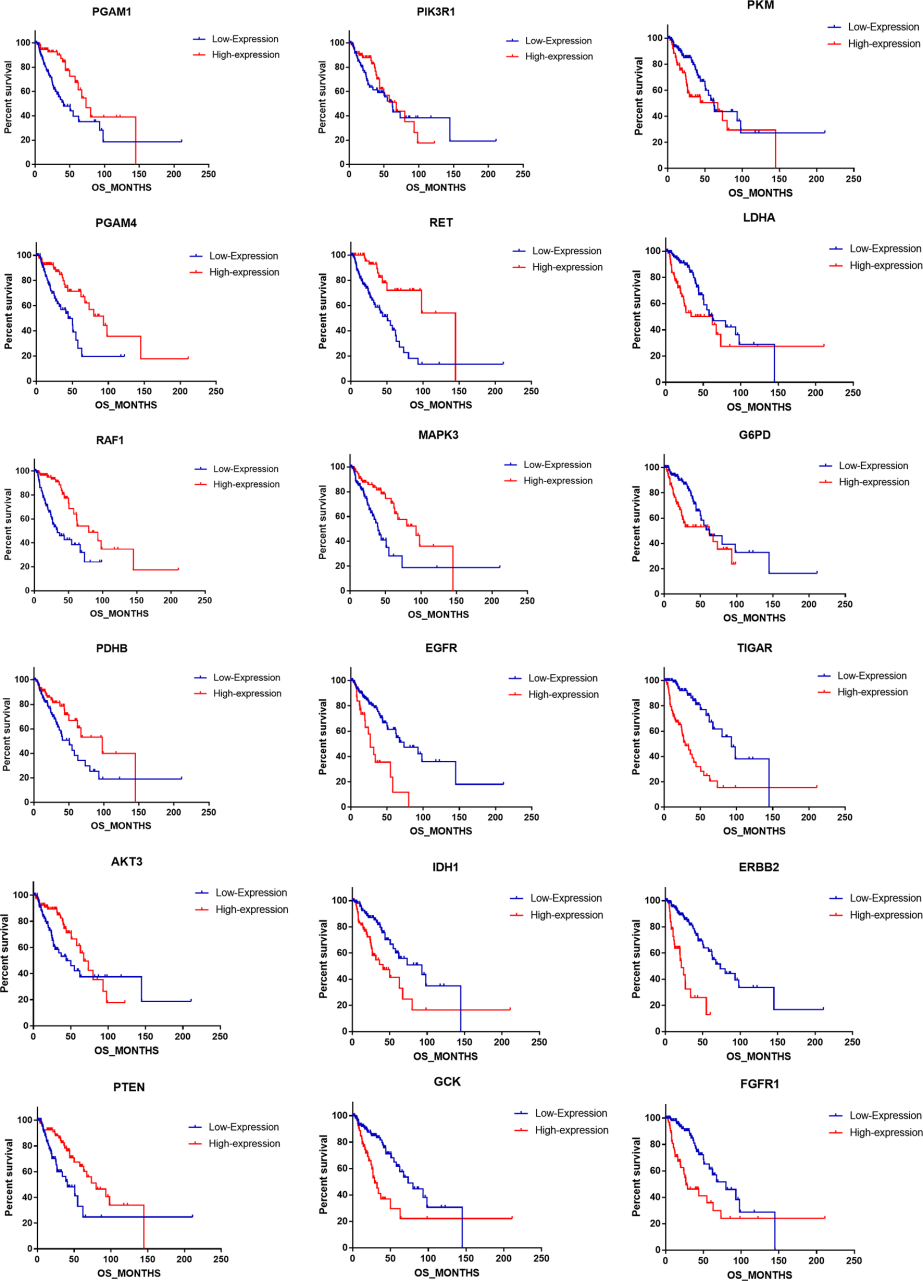
The correlation of singular gene expression difference with the astrocytoma prognosis.

To address the prognostic roles of those survival-related genes, we separately split the astrocytoma patients into two groups according to the single gene expression and additionally compared the prognosis between the two groups (**Figure 5**). Except to *AKT3* and *PIK3R1*, 16 genes showed a significant association with prognosis. Patients with low expression levels of *RET* and *PGAM1* were associated with a greater hazard ratio (HR) for death than that of patients with high expression levels of *RET* and *PGAM1* (*P* < 0.0001). In contrast, patients with high expression levels of *TIGAR*, *ERBB2*, *EGFR*, and *FGFR1* had a higher HR for death than that of patients with low expression levels of those genes (*P* < 0.0001) (**Table 6**).

**Table 6:** The prognostic roles of single metabolism associated gene in astrocytoma.

Gene name	Median survival (mo)		Hazard ratio	95% Confidence interval	P
	Low expression	High expression			
RAF1	33.94	79.93	0.331	1.781–5.128	<0.0001****
RET	50.82	144.94	0.277	2.145–6.069	<0.0001****
EGFR	73.42	26.91	3.007	0.155–0.715	<0.0001****
TIGAR	93.13	29.11	4.276	0.135–0.405	<0.0001****
ERBB2	73.42	21.29	4.301	0.104–0.519	<0.0001****
FGFR1	79.93	26.91	2.776	0.206–0.629	<0.0001****
GCK	73.42	29.11	2.456	0.224–0.739	0.0004***
PGAM4	43.99	93.13	0.424	1.399–3.985	0.0007***
MAPK3	39.72	93.13	0.436	1.362–3.87	0.0011**
PGAM1	41.1	73.42	0.409	1.462–4.094	0.0012**
IDH1	93.13	41.1	2.27	0.256–0.760	0.0012**
PTEN	41.1	79.93	0.475	1.235–3.589	0.0027**
LDHA	62.91	62.12	2.022	0.288–0.850	0.0060**
G6PD	62.91	62.12	1.946	0.299–0.885	0.0089**
PDHB	50.82	98.16	0.54	1.107–3.098	0.0229*
PKM	62.12	67.41	1.685	0.345–1.022	0.0441*
AKT3	43.86	67.41	0.603	0.991–2.776	0.0526
PIK3R1	62.12	67.41	0.802	0.742–2.097	0.4085

*P < 0.05; **P < 0.01; ***P < 0.001; ****P < 0.0001.

**Figure 6:**
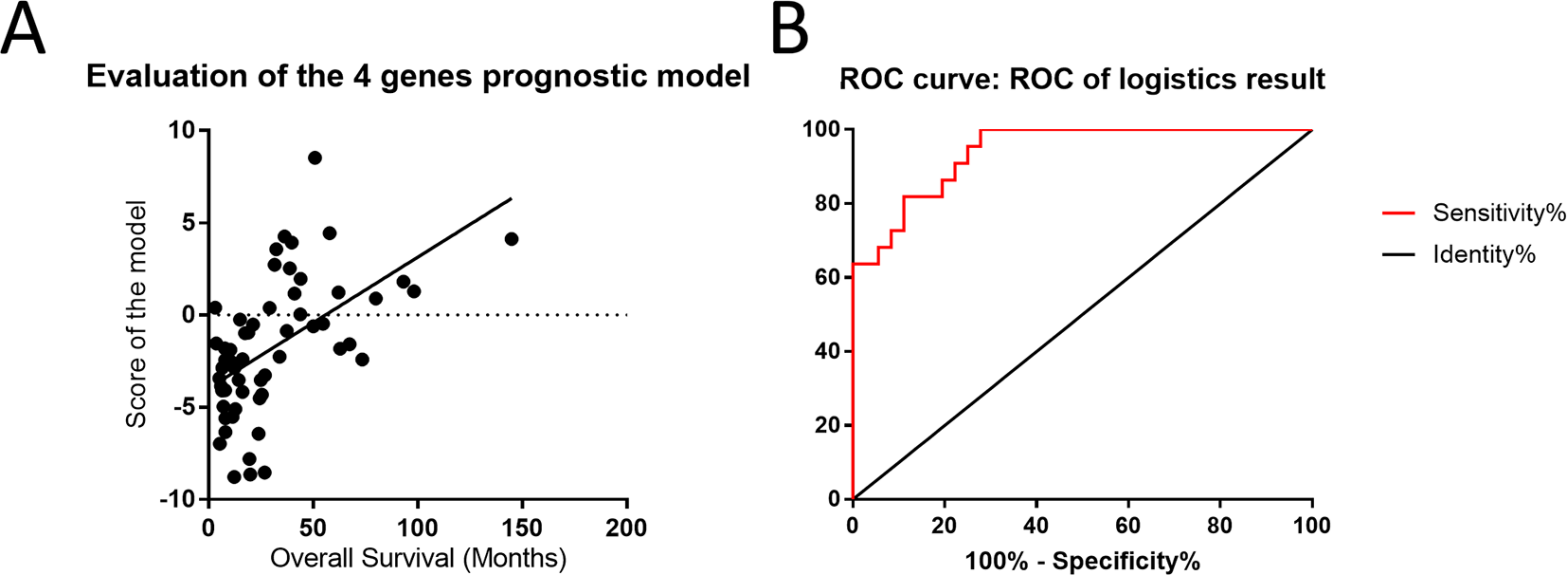
Four-gene panel was utilized to potentially predict the outcomes of patients with astrocytoma. **(A)** The score of the four-gene model including RAF1, AKT3, IDH1, and FGFR1 was positively correlated with overall survival and showed a linearity. **(B)** The area under the receiver operating characteristic curve of the four-gene panel for the prediction of the long- or short-term outcomes of astrocytoma was 0.9407, with a 95% confidence interval of 0.8864 to 0.9949 and *P* < 0.0001.

To evaluate the effects of differences in gene expression on the prediction of the outcome of astrocytoma, we ranked the expression data of 18 genes to construct a regression model. Based on the ranking results, four genes (*RAF1*, *AKT3*, *IDH1*, and *FGFR1*) were independent predictors of the survival status of astrocytoma patients (**Supplementary Table S3**). To integrate these four genes into a single panel, multivariate Cox regression analysis was employed to obtain the coefficient. The risk score was calculated as follows: the risk score was equal to the expression of *RAF1*∗1.801 plus the expression of *AKT3*∗1.545 plus the expression of *IDH1*∗1.569 plus the expression of *FGFR1*∗1.035 (**Figure 6A**). As shown in **Figure 6B**, the area under the receiver operating characteristic curve of the four-gene panel for the prediction of the long- or short-term outcomes of astrocytoma was 0.9407, with a 95% confidence interval of 0.8864 to 0.9949 and a *P* < 0.0001.

## Discussion

Low-grade glioma has complicated characteristics and diverse histologic types. Although the histopathological classification of low-grade gliomas is reliable, it varies between observers and is insufficient to predict clinical outcomes (Louis et al., 2016). Recently, the molecular analysis of tumors has become a critical part of tumor classification and prognostication, and increasing evidence has suggested that defining tumor subtypes based on differences in gene expression in low-grade glioma is meaningful (Verhaak et al., 2010; Eckel-Passow et al., 2015; Louis et al., 2016). In this study, we found that metabolism-associated gene profiling was able to define two primary clusters and four subclusters of patients with low-grade glioma regardless of histologic type. Overall survival differed between the primary clusters and subclusters. We identified 44 genes with significant differences in expression levels between the groups of patients with the worst and best prognoses (**Figure S1**). Some of those genes participate in the regulation of intracellular signal transduction, and others are involved in the metabolism of glucose and other carbohydrates. In addition to the differences in gene expression, we found that the groups had significant differences in histological types, tumor grades, tumor sites, and age. The results showed the specific expression profiles of metabolism-associated genes in patients with low-grade glioma.

Astrocytomas, oligoastrocytomas, and oligodendrogliomas are the three histologic subtypes of low-grade glioma; the subtypes have always been difficult to define according to clinical features (Louis et al., 2014). In the current dataset, patients with astrocytoma had worse prognoses than those of patients with the other two subtypes. We detected the differentially expressed genes in patients with different histological types of glioma, and 45 genes were significantly differentially expressed among the three subtypes. Moreover, 80% of those genes (35 genes) overlapped with the gene set (44 genes) that was associated with different subgroups. Specifically, we determined the expression profiles of metabolism-associated genes in astrocytomas. The results showed that 33 genes had significantly different expression levels, and those differences in expression were closely correlated with OS in patients with astrocytomas. These differences in the expression of metabolism-associated genes not only reveal metabolic differences among the histological subtypes but also suggest that there is metabolic heterogeneity within a single subtype.

In patients with astrocytomas, we identified 11 genes that varied significantly in expression between patients with poor and favorable OS. Additionally, we detected genes with expression levels that were positively and negatively associated with OS, and a correlation existed between the expression levels of these two sets of genes. According to the survival analysis, 16 genes were significantly associated with prognosis. Patients with low expression levels of *RET* and *PGAM1* and high expression levels of *TIGAR*, *ERBB2*, *EGFR*, and *FGFR1* had elevated HRs with regard to survival. The *RET* gene encodes a transmembrane receptor that is a member of the tyrosine protein kinase family of proteins. It has been reported that the mRNA levels of *RET* are elevated in astrocytoma patients with *IDH* mutations, who are known to have prolonged survival (Zhang et al., 2018). *PGAM1* is involved in tumor cell glycolysis and biosynthesis, and this protein had elevated expression levels in high-grade astrocytomas (Liu et al., 2018). Increased expression of these two genes in astrocytomas might inhibit metabolic pathways crucial to the development and progression of tumors.

Low-grade glioma is one of the most malignant human diseases, with a very poor prognosis and scant available information about its biological properties. This study provided new information about the metabolism events affected by the identified genes with differential expression levels. We divided the patients into different subgroups according to their metabolism-associated gene expression patterns. The expression levels of those genes were strongly correlated with the prognosis of patients with astrocytoma, possibly because of their effect on the regulation of the biological behavior of the tumor. This study increases our understanding of the prognostic roles of central carbon metabolism–associated genes in patients with low-grade glioma.

## Data Availability

The datasets analyzed for this study can be found in the cBioPortal for Cancer Genomics (http://cbioportal.org).

## Ethics Statement

Data obtained from the TCGA open-access database was collected from tumors of patients who provided informed consent based on the guidelines from the TCGA Ethics, Law and Policy Group.

## Author Contributions

LZ designed the current study. MG collected the data and performed the statistical test. KW sorted the clinical information and interpreted the result. LW wrote the manuscript. All authors read and approved the final manuscript.

## Funding

This work was funded by the National Natural Science Foundation of China (no. 81702355).

## Conflict of Interest Statement

The authors declare that the research was conducted in the absence of any commercial or financial relationships that could be construed as a potential conflict of interest.
