# Supplementary material for: Prognostic Roles of Central Carbon Metabolism–Associated Genes in Patients With Low-Grade Glioma
Source: Front Genet. 2019 Sep 18;10:831. doi: 10.3389/fgene.2019.00831 (PMC6759923; doi:10.3389/fgene.2019.00831)
Supplement: Supplementary file 1 [file Table_1.docx]

Supplementary Material

# Supplementary Figures and Tables

## Supplementary Figures Tables

**Supplementary Table 1.** Differential expression of metabolic associated genes in each histological type.

| Discrepancy  genes | Astrocytoma （n=194） | | Oligoastrocytoma  (n=130) | | Oligodendroglioma  (n=190) | | Sig. |
| --- | --- | --- | --- | --- | --- | --- | --- |
|  | Mean | SD | Mean | SD | Mean | SD |  |
| NRAS | 0.024475 | 1.173955 | -0.52816 | 0.983008 | -1.03877 | 0.98816 | 6.48E-20 |
| SLC1A5 | -0.01388 | 0.984109 | -0.45732 | 0.632476 | -0.76168 | 0.687491 | 1.05E-17 |
| AKT2 | 0.180944 | 1.428663 | -0.38532 | 1.485506 | -1.09325 | 1.227561 | 2.1E-17 |
| HK2 | 0.373633 | 0.997087 | -0.00828 | 0.972905 | -0.40587 | 0.814396 | 1.47E-14 |
| MTOR | -0.14758 | 1.1465 | -0.47529 | 1.317539 | -1.122 | 1.200789 | 9.36E-14 |
| PFKM | -0.51649 | 1.082464 | -0.02821 | 0.923809 | 0.255715 | 0.910567 | 3.89E-13 |
| PIK3CD | 0.122001 | 1.196367 | -0.19689 | 0.929207 | -0.60409 | 0.703106 | 7.38E-12 |
| SLC16A3 | 0.272198 | 1.156749 | -0.08415 | 0.709759 | -0.27498 | 0.779115 | 6E-08 |
| PIK3R2 | -0.12492 | 0.992558 | -0.00827 | 0.801999 | 0.419726 | 1.090735 | 2.25E-07 |
| GCK | 0.827574 | 1.988237 | 0.035272 | 0.832761 | -0.05416 | 1.7693 | 3.26E-07 |
| AKT3 | -0.26868 | 1.109343 | -0.00909 | 0.86748 | 0.331656 | 1.14207 | 3.62E-07 |
| PGAM1 | -0.56152 | 1.020037 | -0.21665 | 1.040751 | 0.030268 | 1.135734 | 5.57E-07 |
| PIK3R1 | -0.30937 | 0.943479 | -0.04157 | 0.796429 | 0.215713 | 1.077793 | 9.11E-07 |
| GLS2 | -0.2593 | 0.551708 | 0.010205 | 1.000119 | 0.230206 | 1.219623 | 4.68E-06 |
| LDHA | 0.285109 | 1.057771 | -0.04207 | 0.947483 | -0.21946 | 1.017093 | 7.17E-06 |
| PGAM2 | 0.45949 | 1.892816 | -0.04693 | 0.441886 | -0.10911 | 0.576403 | 9.91E-06 |
| KIT | -0.10489 | 1.090395 | -0.09383 | 0.606154 | 0.296394 | 0.921714 | 2.06E-05 |
| RET | -0.07664 | 0.98884 | 0.157421 | 1.10936 | -0.31301 | 0.573722 | 2.53E-05 |
| SIRT3 | -0.53719 | 1.282705 | -0.29918 | 1.152244 | 0.021018 | 1.149108 | 3.74E-05 |
| ERBB2 | 0.252199 | 1.37588 | -0.23352 | 0.392013 | -0.06977 | 0.844853 | 5.64E-05 |
| SLC7A5 | -0.18077 | 0.823835 | -0.1349 | 1.015079 | 0.22291 | 1.063343 | 8.09E-05 |
| G6PD | 0.217011 | 1.235107 | -0.18391 | 0.660091 | -0.12551 | 0.770244 | 0.000132 |
| PTEN | -0.46704 | 1.256328 | -0.33209 | 1.115914 | -0.015 | 1.042304 | 0.000477 |
| PDK1 | -0.15394 | 0.844484 | -0.10168 | 0.637425 | 0.221388 | 1.297673 | 0.000524 |
| FLT3 | -0.20418 | 0.622345 | -0.04822 | 0.549577 | 0.154308 | 1.267274 | 0.000569 |
| HK1 | -0.20734 | 0.741638 | -0.11989 | 0.931998 | 0.151486 | 1.13599 | 0.000771 |
| PGAM4 | -0.21971 | 0.953616 | 0.027484 | 0.944128 | 0.143383 | 1.000749 | 0.001026 |
| PDHB | -0.32448 | 1.070841 | -0.19975 | 1.155827 | 0.085211 | 1.175418 | 0.001603 |
| PIK3R3 | -0.00732 | 0.952206 | -0.30847 | 0.528544 | -0.25773 | 0.961005 | 0.00263 |
| FGFR2 | 0.135779 | 1.069282 | 0.077312 | 0.837711 | -0.18484 | 0.897934 | 0.002721 |
| PIK3CB | -0.19107 | 0.844498 | 0.096929 | 0.940073 | 0.107237 | 1.132135 | 0.004736 |
| FGFR1 | -0.01682 | 1.043093 | -0.24117 | 0.728885 | 0.104702 | 0.967077 | 0.005776 |
| SLC2A2 | 0.172886 | 1.516781 | -0.12101 | 0.299401 | -0.09839 | 0.378503 | 0.006732 |
| EGFR | 1.906888 | 6.850025 | 1.212348 | 5.974023 | 0.325357 | 1.546063 | 0.013249 |
| PDHA1 | -0.14576 | 1.13682 | 0.084857 | 1.059747 | 0.169777 | 1.050545 | 0.01436 |
| PFKP | -0.2026 | 0.871184 | 0.026839 | 0.903982 | 0.066664 | 1.12463 | 0.017296 |
| PIK3CA | -0.10462 | 0.855964 | -0.0136 | 1.007955 | 0.174736 | 1.111333 | 0.020998 |
| PKM | -0.07009 | 0.74966 | -0.20408 | 0.628578 | 0.090867 | 1.30304 | 0.025882 |
| NTRK3 | -0.23237 | 1.01571 | -0.02617 | 1.047034 | 0.030035 | 0.951428 | 0.028478 |
| IDH1 | 0.15466 | 1.071197 | -0.05279 | 0.956629 | -0.09829 | 0.903841 | 0.030399 |
| GLS | -0.14334 | 0.656216 | -0.00173 | 0.982447 | 0.121325 | 1.25225 | 0.033659 |
| FGFR3 | 0.183633 | 1.636539 | 0.085472 | 1.261885 | -0.15267 | 0.867977 | 0.036159 |
| PFKL | 0.091892 | 1.07839 | -0.09518 | 0.901065 | -0.14744 | 0.934356 | 0.046848 |
| MAP2K1 | -0.16804 | 0.769942 | -0.051 | 0.955611 | 0.085287 | 1.239412 | 0.049907 |

**Supplementary Table 2.** Gene expressing variation between poor and good prognosis in astrocytoma.

| Discrepancy  genes | Poor prognosis  (n=59) | |  | Good prognosis  (n=59) | | Sig  (P-value) |
| --- | --- | --- | --- | --- | --- | --- |
|  | Mean | SD |  | Mean | SD |  |
| FGFR1 | 0.8446 | 0.958895 |  | -0.27646 | 0.843808 | <0.001 |
| ERBB2 | 1.465338 | 2.038694 |  | 0.040652 | 0.960407 | 0.001 |
| PGAM4 | -0.8326 | 0.721291 |  | -0.10419 | 1.017005 | 0.003 |
| PGAM1 | -1.36226 | 0.859816 |  | -0.66608 | 0.924901 | 0.004 |
| G6PD | 0.862238 | 1.304473 |  | 0.008745 | 0.881099 | 0.005 |
| RET | -0.57163 | 0.241713 |  | 0.038897 | 1.114217 | 0.006 |
| AKT3 | -0.65949 | 0.775211 |  | -0.01969 | 0.921967 | 0.006 |
| PTEN | -1.18436 | 1.320958 |  | -0.27703 | 1.198585 | 0.008 |
| RAF1 | -0.53177 | 0.909016 |  | 0.081072 | 0.872056 | 0.011 |
| PKM | 0.30439 | 1.169015 |  | -0.21609 | 0.536183 | 0.034 |
| LDHA | 0.934121 | 1.482823 |  | 0.193897 | 1.170278 | 0.039 |

**Supplementary Table 3.** Gene expressing variation between poor and good prognosis in astrocytoma

| Logistics regression | B | SE | Wals | df | Sig. | Exp (B) | 95% CI of EXP (B) | |
| --- | --- | --- | --- | --- | --- | --- | --- | --- |
|  |  |  |  |  |  |  | lower limits | upper limits |
| RAF1 | 1.801 | .832 | 4.682 | 1 | .030 | 6.058 | 1.185 | 30.970 |
| AKT3 | 1.545 | .661 | 5.462 | 1 | .019 | 4.688 | 1.283 | 17.126 |
| IDH1 | -1.569 | .615 | 6.499 | 1 | .011 | .208 | .062 | .696 |
| FGFR1 | -1.035 | .446 | 5.379 | 1 | .020 | .355 | .148 | .852 |
| Constant | .727 | .539 | 1.823 | 1 | .177 | 2.069 |  |  |

## Supplementary Figures


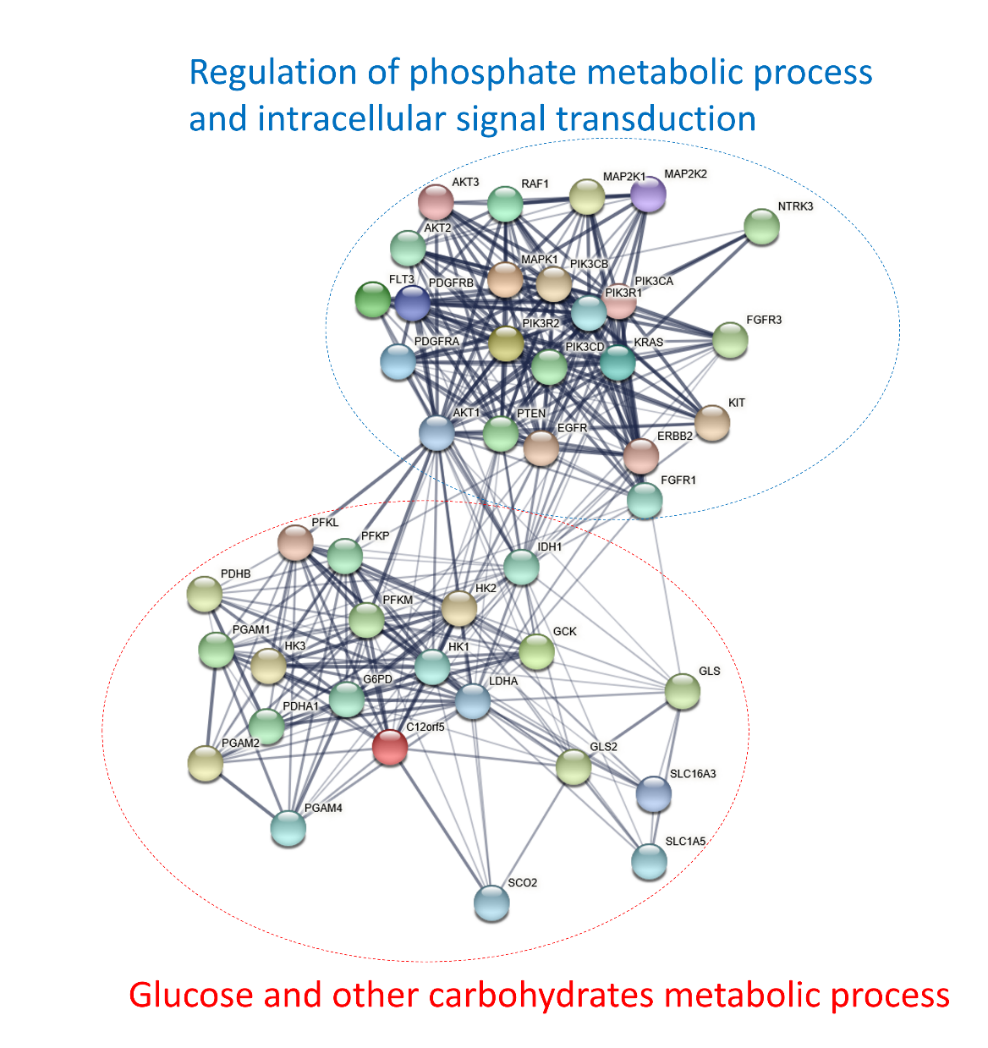


**Supplementary Figure 1.** The GO-analysis showed that among 44 significantly genes prognoses-correlated genes, part of them participate in the regulation of intracellular signal transduction (in the blue circle) and others are particularly involved in the metabolism of glucose and other carbohydrates (in the red circle).
